# Transcranial Direct Current Stimulation Over Motor Areas Improves Reaction Time in Parkinson's Disease

**DOI:** 10.3389/fneur.2022.913517

**Published:** 2022-06-14

**Authors:** Christin M. Sadler, Aline Tiemi Kami, Julie Nantel, Jonathan Lommen, Anthony N. Carlsen

**Affiliations:** ^1^School of Human Kinetics, University of Ottawa, Ottawa, ON, Canada; ^2^School of Rehabilitation Therapy, Queen's University, Kingston, ON, Canada

**Keywords:** Parkinson's disease, tDCS, reaction time, startle, response initiation

## Abstract

**Background:**

Transcranial direct current stimulation (tDCS) has been shown to modulate cortical motor excitability and improve bradykinesia symptoms in Parkinson's disease. It is unclear how targeting different cortical motor areas with tDCS may differentially influence upper limb function for individuals diagnosed with PD.

**Objective:**

This study investigated whether anodal tDCS applied separately to the primary motor cortex and the supplementary motor area would improve upper limb function for individuals with Parkinson's disease. In addition, a startling acoustic stimulus was used to differentiate between the effect of stimulation on motor preparatory and initiation processes associated with upper limb movements.

**Methods:**

Eleven participants with idiopathic Parkinson's disease performed two upper limb simple reaction time tasks, involving elbow extension or a button press before and after either anodal tDCS or sham tDCS was applied over the primary motor cortex or supplementary motor area. A loud, startling stimulus was presented on a selection of trials to involuntarily trigger the prepared action.

**Results:**

Anodal tDCS led to improved premotor reaction time in both tasks, but this was moderated by reaction time in pre-tDCS testing, such that individuals with slower pre-tDCS reaction time showed the greatest reaction time improvements. Startle-trial reaction time was not modified following tDCS, suggesting that the stimulation primarily modulated response initiation processes.

**Conclusion:**

Anodal tDCS improved response initiation speed, but only in slower reacting individuals with PD. However, no differences attributable to tDCS were observed in clinical measures of bradykinesia or kinematic variables, suggesting that reaction time may represent a more sensitive measure of some components of bradykinesia.

## Introduction

Parkinson's disease (PD) is the second most common neurodegenerative disorder in later life, arising from progressive dysfunction of the basal ganglia (BG) and resulting in cardinal motor symptoms, such as resting tremor, rigidity, postural instability, and bradykinesia (1). One of the most debilitating characteristics of PD is bradykinesia, which is typically been defined as slowness of movement, but is also been associated with poor spontaneous movements, decreases in movement amplitude, and delayed reaction times (RT) (2, 3). Although the complete pathophysiology of bradykinesia in PD is not fully understood, bradykinetic symptoms are often attributed to the reduced function of dopaminergic BG outputs that connect to cortical structures associated with motor preparation and action execution (2, 4). The death of dopaminergic neurons in the substantia nigra induces functional impairments between the direct and indirect pathways in the BG, increasing inhibition of the motor thalamic nuclei and subsequently decreasing the excitation of the cerebral cortex (2, 5). Specifically, studies have suggested that hypoactivation in the primary motor cortex (M1) or premotor areas may be the root cause of difficulties in the preparation and initiation of voluntary movements experienced by individuals with PD (2, 6–9). Moreover, it has been shown that hypoactivation in motor areas can result in a delay of the neural activation onset, presenting as increased premotor RT, time-to-peak velocity, and movement duration and variability (4, 10).

Several studies have used neuromodulation interventions with varying degrees of success in an attempt to improve bradykinetic symptoms. To counteract the decrease in neural activation levels in cortical motor areas, such as M1 or supplementary motor area (SMA), resulting from dopamine depletion, several previous studies have attempted to enhance activation using anodal transcranial direct current stimulation (tDCS). tDCS uses a low-level direct current applied over the scalp to induce changes in cortical excitability and neuroplasticity in humans (11). For example, one study showed that, when anodal tDCS was applied over M1 in individuals with PD, motor improvements were observed, including faster RT in a key press response, as well as clinically relevant improvements in the Unified Parkinson's Disease Rating Scale (UPDRS) motor subsection (III) (12). In addition, anodal tDCS over M1 has been associated with decreases in incidences of upper limb freezing (13). However, a recent systematic review and meta-analysis of the effect of tDCS on gait parameters have shown mixed results, in that, while overall small positive effects were evident, more than 50% of the included studies reported no significant effect of tDCS (14). The authors suggested that between-study differences involving medication state, tDCS protocols, and targeted brain regions may have contributed to the differences in reported effects of tDCS.

While many studies have investigated the effect of tDCS applied over M1, few investigations have assessed the impact of tDCS applied over premotor areas such as the SMA, which is involved in many aspects of motor processing, including preparation and initiation of movement (15). Specifically, the role of the SMA in response initiation has been highlighted in studies, showing that firing in many SMA neurons is time locked to the EMG onset (16) and that lesions of the SMA have led to a (transient) akinetic state (17). Hypoactivation in the SMA is present for many individuals with PD (18, 19), and this under-activation has been proposed as a potential source of bradykinetic symptoms (2). In order to counteract activation deficiencies and thus ameliorate motor characteristics of the disease, non-invasive brain stimulation techniques have been proposed as therapeutic interventions. In particular, anodal tDCS has previously been shown to lead to excitability changes in the neural tissue underneath the active electrode, resulting in short-term tonic depolarization (20). When anodal and cathodal tDCS were applied over SMA in healthy individuals, polarity-specific changes in RT were observed, which may be attributable to changes in preparatory activation of the motor system (21). While the effects of anodal tDCS over SMA on multi-limb movements (e.g., gait) for individuals with PD are mixed (22, 23), improvements following anodal tDCS have been reported for some upper-limb tasks (24). Additionally, a recent study from our laboratory has shown that anodal tDCS applied over SMA improved the overall movement production speed of an elbow extension task in individuals with PD, although no effects on RT were observed (25).

It is still somewhat unclear which motor processes are the largest contributors to bradykinesia. For example, it has been suggested that individuals with PD may require more time to complete response preparation processes (26) or that advance information about the required movement (e.g., which limb to use) is simply not used effectively in the initiation of responses (27). Conversely, some researchers have suggested that the bradykinetic features of movement production in PD can be attributed to the malfunctioning BG, which negatively impacts both response preparation *and* response initiation [for a review, see (2)]. One way to potentially distinguish between impairments to each of these processes is through the use of a loud (~120 dB) startling acoustic stimulus (SAS) that can be used as a “synthetic” trigger to involuntarily initiate motor responses that are sufficiently prepared. When the imperative “Go” signal in a simple RT task is replaced by a stimulus that elicits a startle reflex, the intended action is produced much more quickly than in control trials. This RT speeding phenomenon is termed the “StartReact” effect and is believed to result from the involuntary triggering of the pre-planned movement due to startle reflex-related activation in subcortical structures, such as reticular formation and thalamus (28, 29). A few studies have shown that the StartReact effect is intact in individuals with PD, resulting in dramatically shorter RTs and the normalization of bradykinetic movement kinematics compared to movements performed following control tones (30, 31). These results suggest that initiation processes may be the primary locus of RT slowness in PD and can be facilitated by a SAS *via* startle-related activation in subcortical reticulo-thalamic circuits, or indirect activation through the malfunctioning BG (30, 32).

Even though it has been shown that, in PD, the motor response was sufficiently prepared for the SAS to elicit the response, it is still unclear whether preparatory activation in individuals with PD can be further increased following the application of anodal tDCS. It is also unclear whether anodal tDCS applied over different motor regions (e.g., M1 vs. SMA) may differentially impact response initiation latency vs. task-specific kinematics. Thus, the aim of this study was to investigate whether anodal tDCS applied over M1 or SMA facilitates motor response initiation and/or execution in individuals with PD when combined with the presentation of a SAS. Two simple RT tasks using different agonists and different task instructions were tested using the least-affected upper limb to assess whether task complexity interacted with the effects of tDCS and SAS. It was hypothesized that anodal tDCS applied over M1 and SMA would each lead to changes in premotor RT for individuals diagnosed with PD, irrespective of task complexity. Additionally, because of the different neural connections of the motor areas, with subcortical structures including the BG, it was expected that tDCS over SMA would result in greater RT improvements since it is well-reported that SMA plays a role in response initiation processes in PD (2). In contrast, it was hypothesized that greater improvements in bradykinetic symptoms would result from M1 stimulation due to the motor area's predominant role in movement execution. Finally, we hypothesized that, if the response preparation level is significantly increased by tDCS in individuals with PD, then shorter RT latencies would be seen in SAS trials, following tDCS-applied M1 and/or SMA as compared to pre-tDCS.

## Methods

### Participants

In total, 11 right-handed participants (7 males; mean age: 63.5 years; SD: 7.2 years) with diagnoses of idiopathic PD volunteered to participate in the study. An experienced professional performed an initial assessment of all participants using the UPDRS III to verify disease severity. The participants were included if they were presented with mild impairment characterized by scores from 0 to 2 in all items of the UPDRS III (33, 34). This score range was considered a marker of disease progression in order to control for cognitive impairment since the association of cognitive impairment with moderate and severe motor symptoms in PD is well-reported in the literature (35, 36). The participants were excluded if they were being treated with deep brain stimulation (DBS), had dyskinesias, significant tremor in the upper limb on the tested side (to prevent electromyography signal disturbance), significant uncorrected visual and/or hearing impairment(s), or any additional physical upper body conditions that could affect performance on any of the RT tasks. The participants were tested while “ON” their normal anti-parkinsonian medication in order to assess the impacts of tDCS in a realistic sample of individuals living with PD. The characteristics of the participants are provided in Table 1.

**Table 1:** Participant characteristics, including age, sex, disease duration, the least-affected limb, and Unified Parkinson's Disease Rating Scale (UPDRS) motor subsection (III) score.

**Participant**	**Age** **(years)**	**Sex**	**Disease Duration (years)**	**Least Affected Limb**	**UPDRS III Score**
1	48	F	2	L	8
2	60	M	7	R	4
3	60	M	3	R	8
4	64	F	8	L	8
5	57	F	14	L	9
6	73	M	2	L	0
7	71	M	11	R	13
8	69	M	14	R	13
9	61	F	7	L	7
10	67	M	4	R	8
11	68	M	16	R	10
**Mean (SD)**	**63.5 (7.2)**		**8 (5.1)**		**8 (3.7)**

*F, female; M, male; L, left; R, right; SD, standard deviation*.

The study design was a double-blind, randomized crossover, a sham-controlled experiment where, in three separate testing sessions, the participants received stimulation in one of the three tDCS conditions (M1, SMA, and sham). All the participants provided written informed consent after receiving a comprehensive description of all protocols. The sequence of the sessions was randomly assigned using the *random.com* website, and the sequence of conditions for each participant was allocated in a sealed opaque envelope. All the participants and the researcher responsible for the bradykinesia assessments were naïve to the type of stimulation received in each session. All protocols related to the present study were approved by the University of Ottawa Research Ethics Board and were performed in accordance with the seventh revision of the Declaration of Helsinki.

### Bradykinesia Assessment

Upper limb bradykinesia was assessed pre- and post-tDCS in each experimental session using three items selected from the UPDRS III. The three items (numbers 23–25 of the UPDRS III) were finger tapping (FT), hand opening/closing (OC), and pronation/supination (PS) of the hands. These three items were all performed bilaterally and were selected based on functional improvements previously reported in bradykinesia following tDCS (24). Maximum bradykinesia (BK) score of 24 points was possible by summing a score of 0–4 for the three items with the right (R) and left (L) limb components (see Equation 1).


(1) $$\begin{array}{c} \text{BK SCORE}=FTR+FTL+OCR+OCL+PSR+PSL \end{array}$$

The researcher responsible for assessing the three items from the UPDRSIII was trained by an experienced professional, and the scores of the first five participants were compared between the researcher and the experienced professional to ensure similar ratings. Bradykinesia assessments were performed by a separate researcher from the one administering the tDCS to maintain assessment blinding to the tDCS condition.

### Apparatus and Tasks

**Figure 1:**
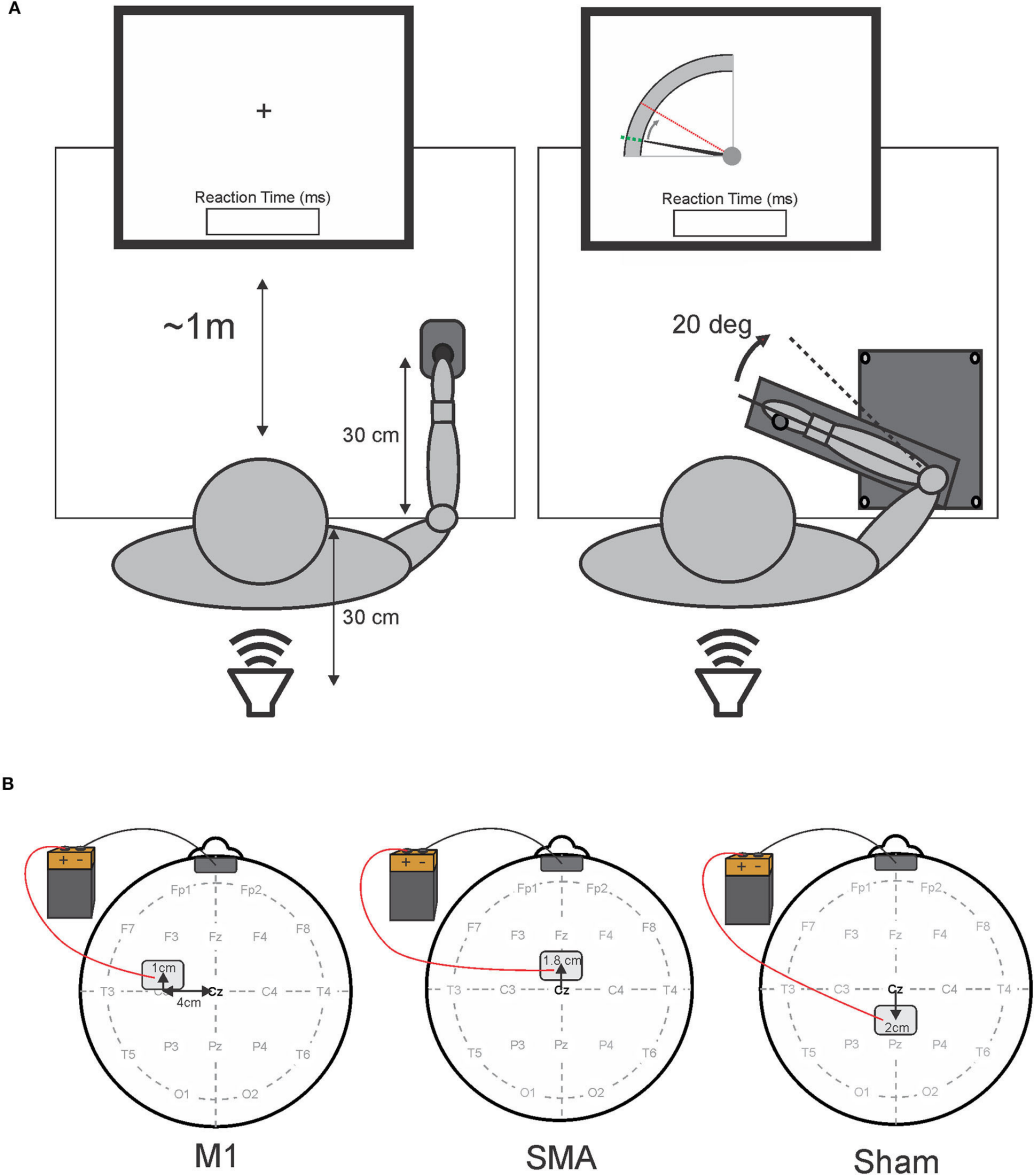
Schematic representation of experimental tasks **(A)** and tDCS electrode placement **(B)**. **(A)** Shows a schematic overhead representation of the experimental apparatus and participant placement. The left side depicts the button-press task with the fixation cross, while the right side depicts the elbow-extension task, and the visual feedback on target accuracy presented to the participant at the beginning and the end of each trial for the elbow-extension task. The position of the elbow was represented by the solid black cursor moving from the home position (a dotted green line) to the 20° target (a dashed red line). In both tasks, reaction time feedback was displayed immediately following each trial. A loud speaker placed 30 cm behind the participants delivered the auditory stimuli. Panel B depicts tDCS electrode placement for each session. The left side illustrates primary motor area (M1) stimulation (note: the anode electrode was placed over M1 contralateral to the limb used to perform the reaction time tasks); the center image illustrates supplementary motor area (SMA) stimulation; the right side illustrates sham stimulation. The cathode electrode was placed on the forehead (center, above the eyebrows) for all sessions.

The participants were seated upright in a comfortable chair, approximately at a 1 m distance from a 24-inch LCD computer screen (Asus VG248, 144 Hz refresh), and used their least-affected upper limb (self-reported) to perform two simple RT tasks with different movement precision requirements. A depiction of each task is presented in Figure 1A. The first task was a button-press task, which required the participants to simply press a telegraph key (Ameco AM-K4B) as quickly as possible, following an auditory go-signal. The telegraph key was fixed 30 cm in front of the participant with the hand resting on top of the key (left panel, Figure 1A). The second task required the participants to perform targeted elbow extension as quickly as possible, following an auditory go-signal. The participants grasped a handle of a custom-made aluminum manipulandum that moved in the horizontal plane with an axis of rotation about the elbow. The home position was set at 90° flexion of the elbow with the shoulder flexed and abducted 30°. The participants were instructed to perform a targeted 20° extension movement of the elbow from the home position to a visual target shown on the screen (right panels, Figure 1A). This external visual cue disappeared following the warning signal and reappeared after each trial to provide visual feedback on target accuracy.

For both tasks, feedback was removed at the onset of each trial, and a fixation cross was presented, followed by an auditory warning “Get Ready” signal, consisting of a 100 ms, 200 Hz tone. After a randomly generated foreperiod of 2,000–2,500 ms, the imperative “Go” stimulus was presented (1 kHz tone; 100-ms duration; 80 dB). On a selection of trials, a SAS (20–20,000 Hz; white noise pulse; 25-ms duration; 120 dB) replaced the control tone. The warning signal was delivered by a computer speaker, while the imperative stimulus was generated with digital-to-analog hardware (National Instruments PCIe-6321), amplified, and presented by a loudspeaker (MG Electronics M58-H; frequency response, 300 Hz−11 kHz; rise time <1 ms), which is located 30 cm behind the participant. Stimulus intensity was confirmed using a precision sound level meter (Cirrus research CR: 162C, A-weighted). Following each trial in both tasks, the participants received visual feedback on their RT displayed for 3,500 ms until the beginning of the next trial. A customized LabVIEW (National Instruments Inc.) program controlled the timeline for each trial and the display of RT information to the participant.

### Procedure

Following the bradykinesia assessment using the UPDRS III, the participants were asked to perform 10 practice trials, followed by one block of 20 testing trials for the button-press task. The participants then performed 10 practice trials, followed by one block of 20 testing trials for the elbow extension task. Both tasks were performed using the least-affected limb to minimize any impact of tremor on EMG recordings. In each task, the testing block of 20 trials consisted of 15 control trials, and five SAS trials pseudo-randomly dispersed among the control trials, where neither two consecutive trials nor the first three trials were SAS trials (37). Practice blocks contained no SAS trials.

After the completion of the two simple RT tasks, anodal tDCS was applied over either M1 or SMA, or sham stimulation was applied (see tDCS protocol and Figure 1). Bradykinesia was immediately reassessed following the end of stimulation, using the UPDRS III before another (post-tDCS) block of 20 testing trials was performed for each of the RT tasks.

### tDCS Protocol

Following recommended procedure and safety guidelines, two electrodes, each consisting of a flexible conductive carbon insert inside of a sponge electrode (Soterix EASYpads; 15 cm^2^), were placed on the scalp of the participants for stimulation of a targeted brain area (38). To identify electrode placement sites for each participant, the midpoint between the nasion and inion, and the left and right preauricular notches were first identified (position Cz in the international 10–20 system) and used as a landmark of origin to locate the M1, SMA, and sham stimulation sites. A depiction of electrode placement for each stimulation session is presented in Figure 1B. In order to target M1, the active (anodal) electrode was positioned by measuring 4 cm lateral (contralateral to the tested limb) and 1 cm anterior to the origin (left panel, Figure 1B). For SMA stimulation, the active electrode was positioned 1.8 cm anterior to the origin (center panel, Figure 1B). This location of the SMA has also been previously confirmed with the use of transcranial magnetic stimulation (39). For sham stimulation, the “active” electrode was placed 2 cm posterior to the origin (right panel, Figure 1B). In all stimulation sessions, the return (cathode) electrode was placed on the center of the forehead directly above the eyebrows (Figure 1B). Each electrode was gently secured to each stimulation location with standard foam under-wrap tape to ensure the electrode's position did not change during the session.

A Soterix Medical 1- × -1 tDCS Model 1300A Low-Intensity Stimulator was used to deliver the electrical current. Stimulation was set at an intensity of 1.5 mA and applied for 10 min during anodal stimulation to M1 and SMA. For sham stimulation, the “auto-sham” function was selected on the device. This function automatically ramps up the current to 1.5 mA (for about 30 s) and back down to 0 at the beginning and the end of the designated stimulation period (10 min) without the participant's awareness. In this way, some participants might have felt an initial sensation of the stimulation, but they did not receive active tDCS for the rest of the stimulation period. The stimulation sessions (M1, SMA, and sham) were conducted at least 48 h apart to ensure a complete washout of any residual tDCS effects (40, 41).

### Data Acquisition

A wireless electromyography (EMG) system (Delsys Trigno) was used to record muscle activity. EMG data were collected from the lateral head of the triceps brachii, the biceps brachii, and the flexor carpi radialis (FCR) muscles in each participant's least-affected upper limb (see Table 1). In addition, EMG data were collected from the sternocleidomastoid muscle (SCM) contralateral to the upper limb tested to monitor the startle reflex. The Delsys Trigno surface electrodes were placed in the middle of the muscle bellies, aligned parallel to the muscle fibers, and attached with double-sided adhesive strips. Before the application of the electrodes, the skin surface below each recording site was cleaned with alcohol swabs, and a conductive gel was applied to decrease electrical impedance. Raw band-passed (20–450 Hz) EMG data were digitally sampled at 4,000 Hz using a customized LabVIEW program and stored for offline analysis. Data collection was initiated by the computer for each trial 1 s prior to the presentation of the imperative “Go” stimulus and continued for 3 s.

### Data Reduction and Analysis

Trials were discarded in cases where the participants anticipated the go-signal (RTs shorter than 50 ms; 80 trials), did not pay sufficient attention to the task (RTs longer than 500 ms; 20 trials), or performed movement errors (e.g., no button press or multiple component movements; 58 trials). Thus, a total of 158 trials (including 30 SAS trials) were discarded out of 2,640 trials (retention rate of 94%). A startle reflex-related burst of EMG activity in SCM is considered to be a robust and reliable indicator that a startle reflex was elicited (42). SCM activation was defined as an EMG burst onset, occurring within 25–120 ms of presentation of the SAS. SAS trials where no startle activity was observed in SCM (SCM–; 257 trials) were categorized separately from those in which SCM activity was observed (SCM+; 373 trials). This enabled the impact of the “startle” to be assessed while controlling for the effect of the loud stimulus (42).

Premotor RT, which is defined as the time between the auditory go-signal or SAS and the EMG burst onset in the agonist for each task (FCR for the button-press task, and triceps brachii for the elbow extension task), was the primary dependent variable for both the button-press and elbow-extension tasks. The EMG burst onset for each muscle was defined as the point where rectified and filtered EMG activity reached two SDs above the baseline level and remained elevated for at least 20 ms. To verify the onset points, EMG traces were displayed on a computer monitor along with EMG onset markers computed using a custom LabVIEW program and then manually adjusted if necessary to correct for any possible errors due to the strictness of the algorithm [see (43)].

Kinematic variables were only analyzed for the elbow extension task as the information from the button-press was limited to the button being either on or off. As such, for the button-press task, only the movement onset was available as indicated by the closing of the switch. For the elbow extension task, kinematic variables included the movement onset, peak velocity, time-to-peak velocity, peak displacement, and time-to-peak displacement. The movement onset was identified as the first point of change of more than 0.2 deg of angular displacement from the home position, following the “Go” signal. Peak velocity was defined as the maximum angular velocity achieved prior to reaching peak displacement. Time-to-peak velocity was the time between the movement onset and the peak velocity. Peak displacement was defined as the maximum angular displacement attained between the movement onset and final position. Time-to-peak displacement was the time between the movement onset and peak displacement.

### Statistical Analysis

Individuals with PD present with heterogeneous and idiosyncratic motor and non-motor symptoms (44, 45). As such, to assess whether tDCS had a differential effect on fast vs. slow responders, mean pre-tDCS RT from control trials for the button press and arm extension tasks (in each tDCS session) was included as a moderating factor in the RT analyses, as pre-tDCS RT was expected to accurately reflect baseline movement initiation speed. Similarly, the pre-tDCS BK score was included as a factor in the analyses of kinematic variables as the BK score primarily assessed movement execution speed.

Primary dependent variables were analyzed using linear mixed effects (LME) analyses in R statistical software (46). LME models were used as they allow all data points to be retained without violating assumptions of independence. Analysis of premotor RT was carried out using separate models for control and SAS trials to determine if tDCS impacted RT, depending on the task for each stimulus condition. As such, in the LME analysis of control trials, tDCS Session (M1, SMA, Sham), time (pre-tDCS, post-tDCS), task (elbow extension, button press), and mean pre-tDCS RT were specified as interacting fixed factors with the participants specified as a random factor [e.g., model = premotor RT ~ Session ^*^ Time ^*^ Task ^*^ Mean Pre-tDCS RT + (1 | Participant)]. For SAS trials, session, time, task, mean pre-tDCS RT, and SCM presence (SCM+, SCM–) were specified as interacting fixed factors with the participants specified as a random factor.

Analysis of elbow extension task kinematics was carried out separately for each variable using tDCS Session, time, auditory stimulus (Control, SAS SCM+, SAS SCM–), and BK Score as interacting fixed factors with the participants specified as a random factor. The auditory stimulus was included in the kinematics analyses since the effect of the stimulus on several kinematic measures has been previously reported (30). Because similar kinematic variables (e.g., peak velocity) were not available for both the button-press task and the elbow extension task, analysis of kinematic variables was limited to the elbow extension task.

The mean proportion of SAS trials in which a short latency burst of EMG activity in SCM was observed was corrected for normality using an arcsine square root transformation and analyzed using a 3-Session- × -2-Time- × -2-Task repeated measures ANOVA. Similarly, upper limb bradykinesia (BK) scores were corrected for normality using a square root transformation and analyzed using a 3-Session- × -2-Time repeated measures ANOVA.

For all analyses, any significant effect of the tDCS session was only considered to be meaningful if there was also a significant interaction with time (i.e., revealing differences in pre-tDCS and post-tDCS measurements that were different from sham). The significance value for all statistical tests was set at *p* < 0.05. Data used in LME models were examined for homoscedasticity and the approximate normal distribution of residuals and were scanned for influential cases. A Bonferroni correction was applied to correct for multiple LME models per dependent variable. Participants' age, sex, and disease duration were initially included in the LME analyses; however, because none of these factors significantly improved any of the models, they were removed. LME analyses were performed in R (46) using the lme4 package (47) along with the lmerTest package (48) to provide *p*-values. Estimated marginal means, estimated slopes, and pairwise comparisons between means and between slopes, were carried out using the emmeans package (49) with the Tukey method applied to correct for multiple comparisons.

## Results

### Premotor RT

#### Control (Non-SAS) Trial RT

**Figure 2:**
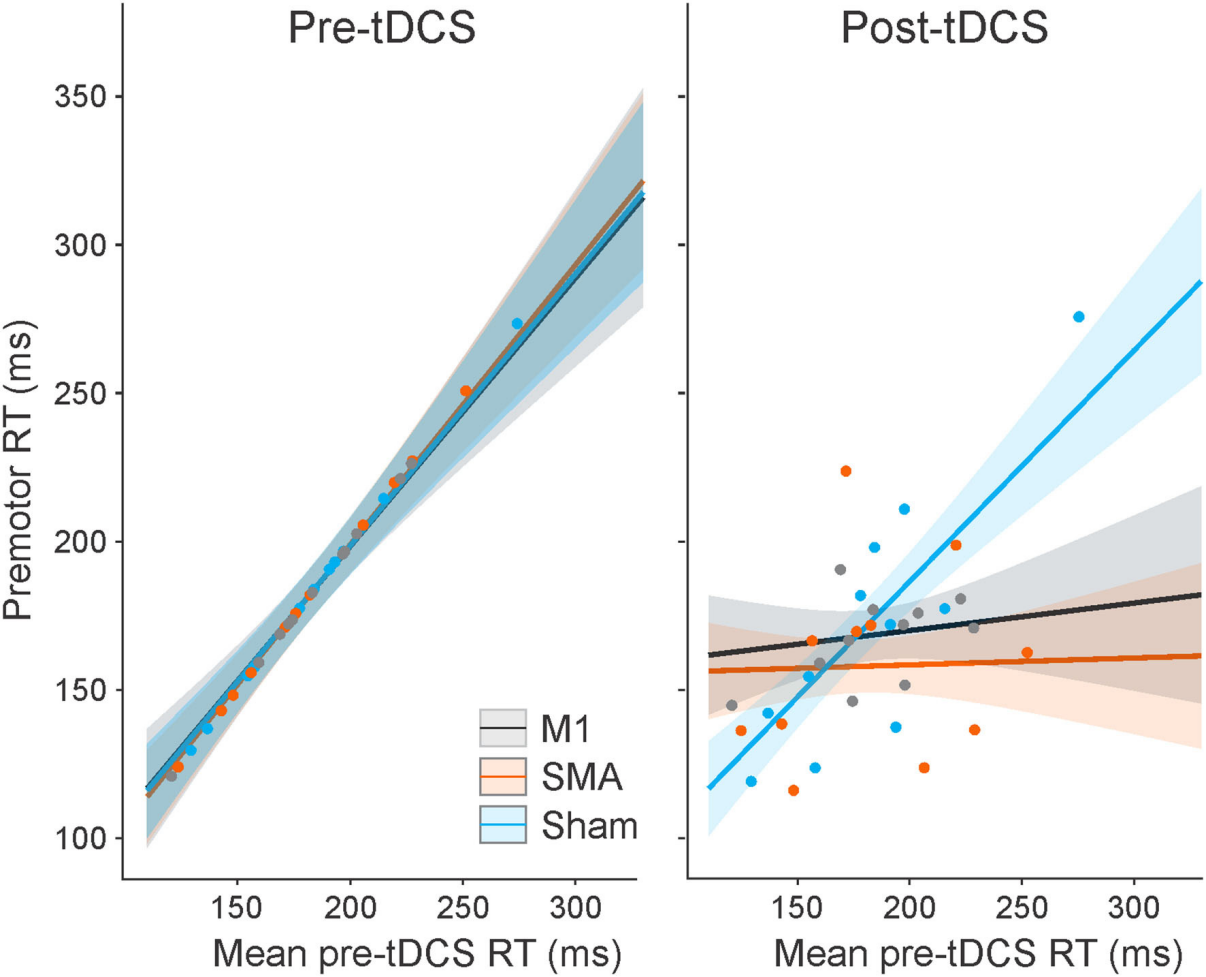
Model-fitted predicted control trial premotor reaction time (RT) collapsed across tasks as a function of mean pre-tDCS control RT for each tDCS session (M1 = a black line; SMA = an orange line; sham = a blue line). The left panel shows pre-tDCS data, and the right panel shows post-tDCS. Shaded areas represent 95% CI. Dots represent participant mean RT values in each tDCS session (note that these means are collapsed across tasks with different pre-tDCS mean RT values, and the models take all individual data points into consideration).

The analysis of control trial premotor RT indicated that there was no main effect of the task (elbow extension vs. button press), *F*_(1, 1664.2)_ = 1.633, *p* = 0.201, as well as no significant interactions involving the task (all *p*-values >0.196). On the other hand, all other main effects and interactions were significant (all *p*-values < 0.001), and, as such, were superseded by the three-way interaction between session, time, and mean pre-tDCS RT [*F*_(2, 1818.1)_ = 10.105, *p* < 0.001]. This interaction is illustrated in Figure 2 (collapsed across tasks). *Post hoc* analysis looked at how RT (as a function of pretest RT) changed from pre-tDCS to post-tDCS for each session. This analysis revealed that, in the pre-tDCS testing, the slope of the increase in RT as a function of mean pretest RT was very close to 1 for all three tDCS sessions (Figure 2, left panel), and there was no significant difference in slopes between the sessions (*p*-values >0.999). However, there were significant differences between pre-tDCS and post-tDCS slopes following M1 stimulation [pre = 0.906, SE = 0.127; post = 0.093, SE = 0.126; slope difference = −0.814, SE = 0.167; *t*_(1,818)_ = 4.863, *p* < 0.001] and following SMA stimulation [pre = 0.945, SE = 0.097; post = 0.023, SE = 0.102; slope difference = −0.922, SE = 0.130; *t*_(1,822)_ = 7.105, *p* < 0.001], whereby premotor RT was impacted to a larger degree in the participants with longer (slower) pre-tDCS RT (Figure 2, right panel). On the other hand, there was no difference in the RT slope following sham stimulation [pre = 0.918, SE = 0.100; post = 0.778, SE = 0.102; slope difference = −0.140, SE = 0.131; *t*_(1,818)_ = 1.071, *p* = 0.893]. Finally, in the post-tDCS testing, the RT slopes were significantly different than sham for both M1 stimulation [slope difference = −0.686, SE = 0.157; *t*_(1,576)_ = 4.369, *p* < 0.001] and SMA stimulation [slope difference = −0.755, SE =0.134; *t*_(1,825)_ = 5.628, *p* < 0.001], but not different from one another [slope difference = −0.069, SE = 0.159; *t*_(1,456)_ = 0.437, *p* = 0.998].

#### SAS Trial RT

**Figure 3:**
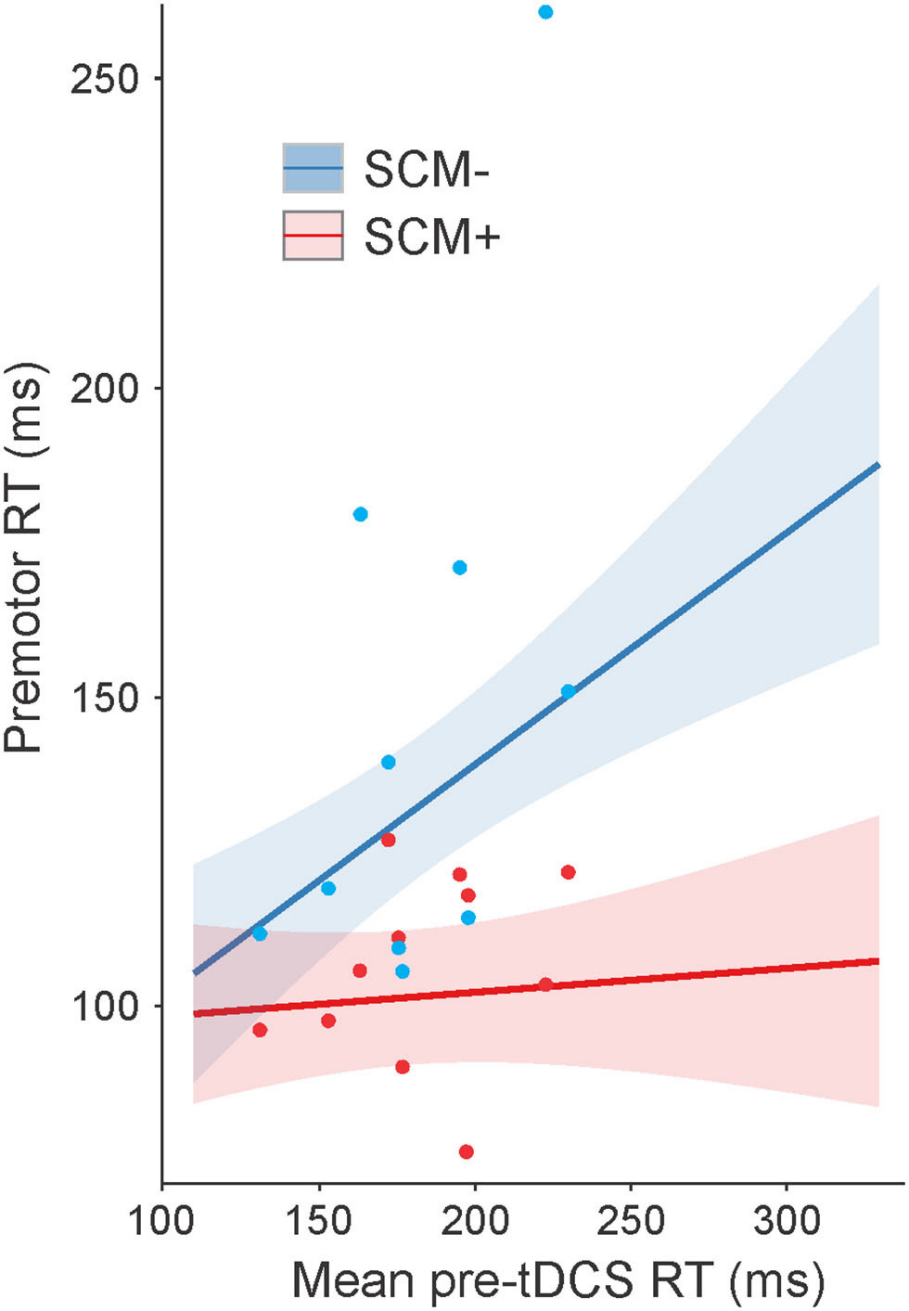
Model-fitted predicted premotor reaction time (RT) on startle (SAS) trials collapsed across tasks as a function of mean pre-tDCS control trial RT. The red line shows predicted RT for trials where a startle reflex in sternocleidomastoid (SCM) was observed (SCM+), and the blue line shows predicted RT for trials where a startle reflex was not observed (SCM–). Shaded areas represent 95% CI. Dots represent participant mean RT values (note that these means are collapsed across tasks with different pre-tDCS mean RT values, and the model takes all individual data points into consideration).

For RT on SAS trials, a significant main effect was found for mean pre-tDCS RT, *F*_(1, 477.9)_ = 10.159, *p* = 0.002, but this was superseded by a significant interaction between pre-tDCS RT and SCM presence, *F*_(1, 580.4)_ = 10.748, *p* = 0.001. This interaction is illustrated in Figure 3 (collapsed across tasks). The steepness of the slope of RT increase, as a function of pre-tDCS RT, was much smaller on SCM+ trials compared to SCM− trials [SCM+ = 0.039, SE = 0.071; SCM– = 0.374, SE = 0.094; slope difference = −0.335, SE = 0.102; *t*_(580)_ = 3.272, *p* = 0.001]. Although there was a main effect of tDCS session on RT, *F*_(2, 579.6)_ = 6.135, *p* = 0.002, it did not interact with time, *F*_(2, 573.27)_ = 0.680, *p* = 0.507. Similar to control trial RT, there was no main effect of task on SAS RT, *F*_(1, 1664.2)_ = 1.633, *p* = 0.201, and no significant interactions involving the task, and all other main effects and interactions were not significant (all *p*-values ≥0.086) or not meaningful (as described in Statistical analysis).

### Kinematic Measures (the Elbow- Extension Task Only)

#### Peak Velocity and Time-to-Peak Velocity

**Figure 4:**
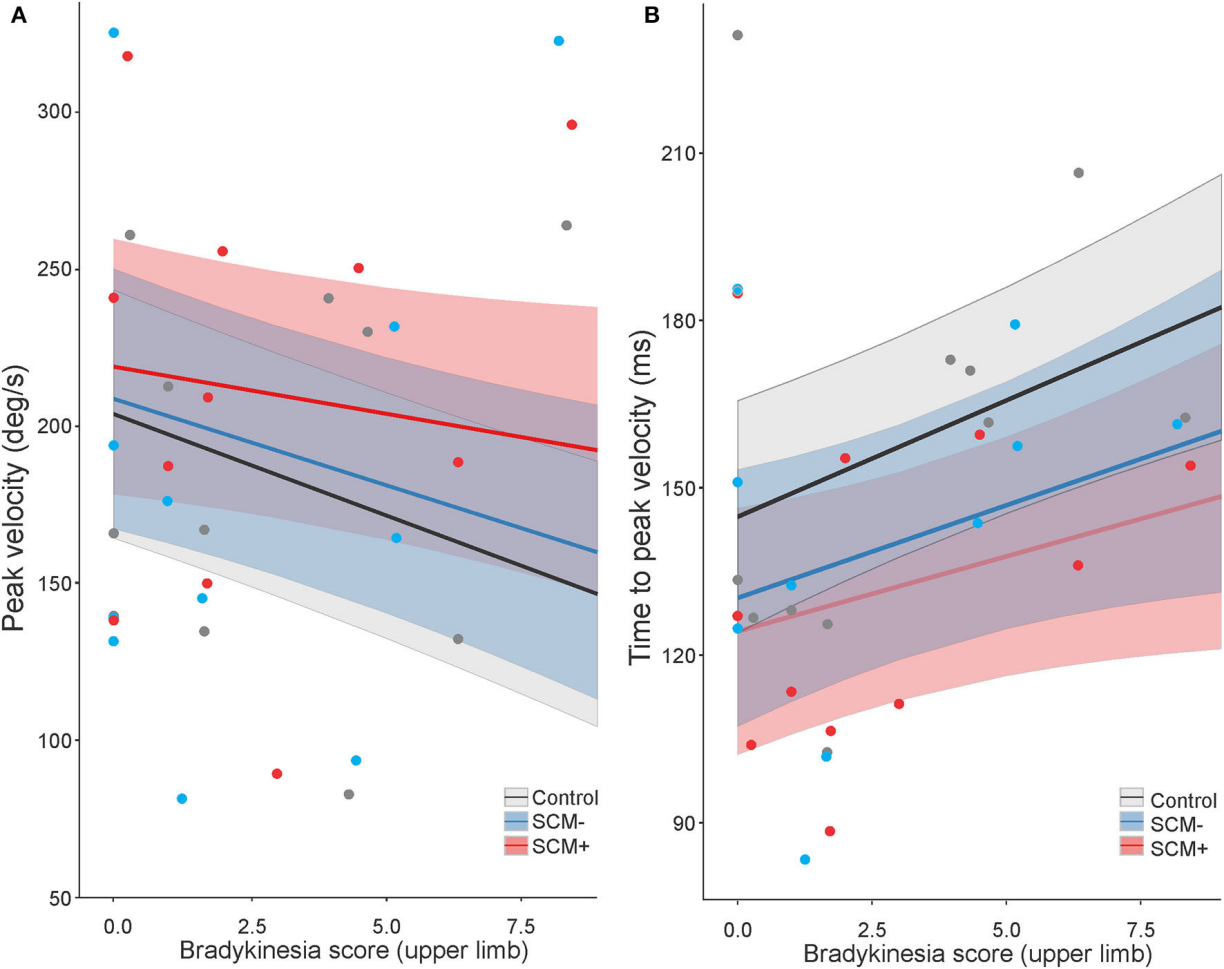
Model-fitted predicted peak velocity **(A)** and time-to-peak velocity **(B)** for the elbow-extension task as a function of a bradykinesia score. The black lines show predicted values for control trials, the red lines show predicted values for startle trials where a startle reflex in sternocleidomastoid (SCM) was observed (SCM+), and the blue lines show predicted values for startle trials where a startle reflex was not observed (SCM–). Shaded areas represent 95% CI. In all auditory stimulus conditions, higher bradykinesia scores predicted lower peak velocities **(A)** and longer time-to-peak velocity **(B)**. SCM+ trials predicted higher peak velocities than SCM– trials and control trials **(A)**, whereas both SCM+ and SCM– trials predicted shorter time to peak velocity compared to control trials **(B)**. Dots represent participant mean values (note that these means are collapsed across tDCS sessions, which may have had different BK scores, and the model takes all individual data points into consideration).

For peak velocity, a significant main effect was found for the BK score, *F*_(1, 1125.2)_ = 10.944, *p* = 0.001, and for auditory stimulus, *F*_(2, 1188.5)_ = 3.359, *p* = 0.035. These main effects are depicted in Figure 4A, indicating that the participants with a higher BK score tended to exhibit lower peak velocities. *Post hoc* tests also confirmed that when collapsed across all other factors, SCM+ trials showed higher peak velocities (210 deg/s, SE = 20.) than control trials [185 deg/s, SE = 19.8; *t*_(1,189)_ = 6.175, *p* < 001] and SCM– trials [193 deg/s, SE = 20.3; *t*_(1,190)_ = 2.796, *p* = 0.015]. There was no significant difference in peak velocity between control and SCM– trials, *t*_(1,189)_ = 1.485, *p* = 0.299. For peak velocity, there was also a significant interaction between tDCS session and time, *F*_(2, 1188.)_ = 4.247, *p* = 0.015; however, *post hoc* multiple comparisons did not show any significant differences between pairs of interest (i.e., pre-tDCS vs. post-tDCS for each session; all *p*-values >0.415).

Time-to-peak velocity also showed significant main effects for both the BK score, *F*_(1, 808.2)_ = 8.573, *p* = 0.004, and auditory stimulus, *F*_(2, 1201.4)_ = 11.791, *p* < 001. These main effects are depicted in Figure 4B, indicating that the participants with a higher BK score tended to exhibit increased time-to-peak velocity. Furthermore, *post hoc* tests confirmed that control trials showed longer time-to-peak velocity (157 ms, SE = 10) as compared to SCM+ trials [132 ms, SE = 10.4; *t*_(1,202)_ = 7.706, *p* < 001] and SCM– trials [140 ms, SE = 10.7; *t*_(1,203)_ = 4.134, *p* < 001]. There was no significant difference in time-to-peak velocity between SCM+ and SCM– trials, *t*_(1,204)_ = 1.568, *p* = 0.260. Similar to what was seen in peak velocity, time-to-peak velocity also showed a significant interaction between tDCS session and time, *F*_(2, 1200.2)_ = 3.722, *p* = 0.024; however, after correcting for multiple comparisons, there were no significant differences between any of the pairs of interest (*p*-values >0.388).

#### Peak Displacement and Time-to-Peak Displacement

**Figure 5:**
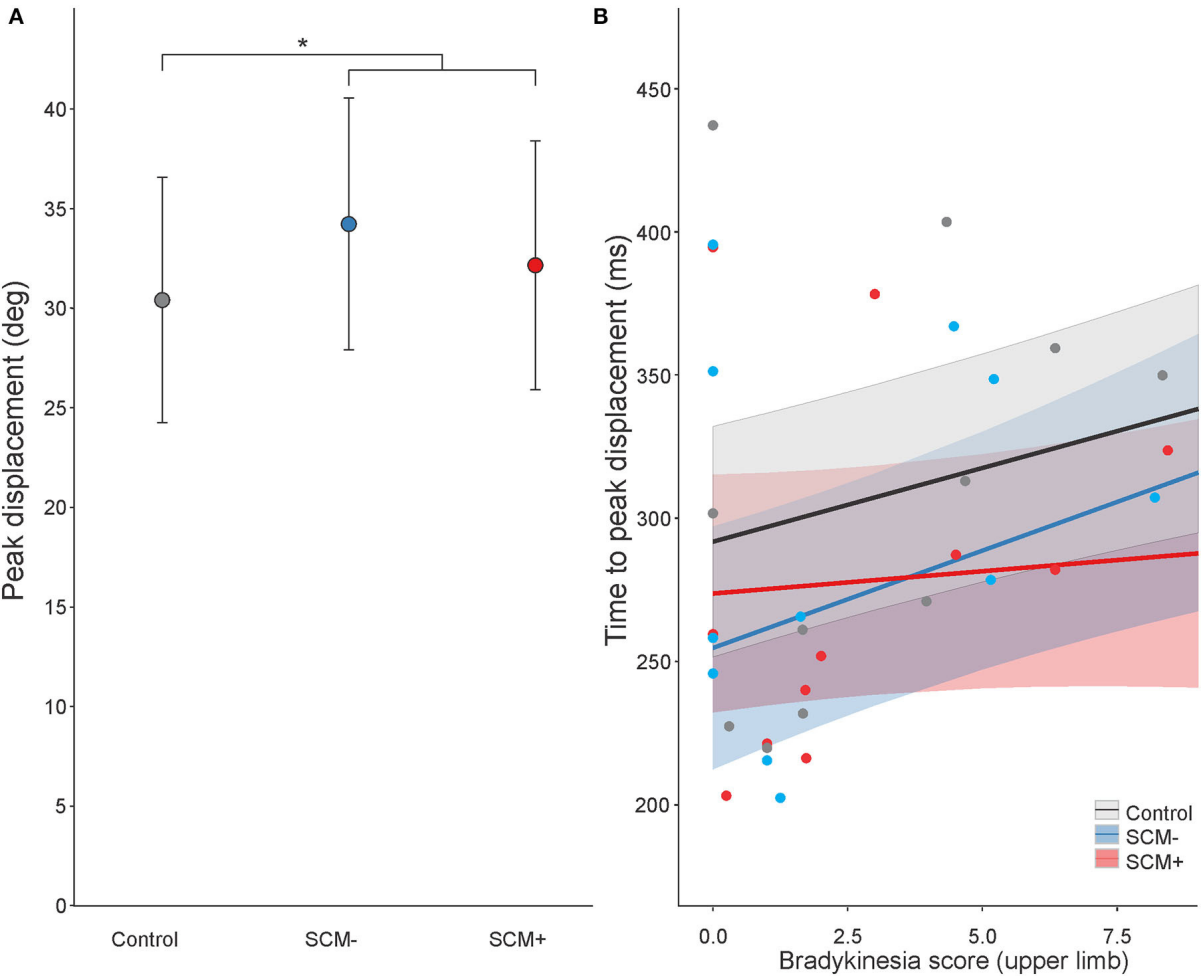
Estimated marginal means (95% CI) for peak displacement as a function of stimulus condition **(A)** and model-fitted predicted time-to-peak displacement as a function of a bradykinesia score **(B)**. For time-to-peak displacement, the black line shows predicted values for control trials, the red line shows predicted values for startle trials where a startle reflex in sternocleidomastoid (SCM) was observed (SCM+), and the blue line shows predicted values for startle trials where a startle reflex was not observed (SCM–). Shaded areas represent 95% CI. Dots represent participant mean values (note that these means are collapsed across tDCS sessions, which may have had different BK scores, and the model takes all individual data points into consideration).

Peak displacement Showed a significant main effect of auditory stimulus, *F*_(2, 1188.3)_ = 6.629, *p* = 0.001), which is depicted in Figure 5A. *Post hoc* tests confirmed that, compared to control, significantly larger peak displacement was measured on SCM+ trials, *t*_(1,189)_ = 3.259, *p* = 0.003, and, on SCM– trials, *t*_(1,189)_ = 4.846, *p* < 0.001. Peak displacement between SCM+ and SCM– was not significantly different, *t*_(1,190)_ = 1.853, *p* = 0.153. In addition, there was a significant interaction between tDCS session, time, and BK score, *F*_(2, 1187.9)_ = 3.632, *p* = 0.027. While it appears the interaction may have been driven by a positive slope in the post-tDCS trials following SMA stimulation, Tukey corrected *post hoc* comparisons of the slopes revealed no significant differences between any of the pairs (all *p*-values >0.071).

For time-to-peak displacement, there was a main effect of auditory stimulus, *F*_(2, 1200.6)_ = 14.648, *p* < 0.001, and a main effect of BK score, *F*_(1, 1119.8)_ = 8.206, *p* = 0.004, but these were superseded by a significant interaction between the factors, *F*_(2, 1201.2)_ = 3.338, *p* = 0.036 (Figure 5B). *Post hoc* tests of the slopes confirmed that the increase in time-to-peak displacement as a function of BK score on SCM+ trials (1.56 ms/point, SE = 2.04) was significantly smaller, *t*_(1,201)_ = 2.353, *p* = 0.049, compared to control (5.14 ms/point, SE = 1.52). The increase in time-to-peak displacement on SCM– trials (6.79 ms/point, SE = 2.36) was not different from SCM+ (*p* = 0.068) or control (*p* = 0.672).

### Percentage of SCM+

The percentage of SAS trials that resulted in an observed startle reflex-related burst of EMG activity in the SCM was 60.1% (SD = 27.6, range = 41.1–100%). This incidence did not differ between tasks, tDCS sessions, or time, and there were no significant interactions between the factors (all *p*-values >0.254).

### Bradykinesia Score

Bradykinesia scores (described in the Bradykinesia assessment) were compared between pre-tDCS and post-tDCS for all three stimulation sessions. There were no significant main effects or interactions between any of the factors (all *p*-values >0.069).

## Discussion

The purpose of the present study was to examine whether the RT and kinematic characteristics of voluntary movements in individuals with PD could be improved by anodal tDCS and whether any interactions existed between the stimulation site of tDCS, the complexity of movement, and the presentation of a SAS. In order to account for heterogeneity in bradykinesia between individuals with PD, RT analyses included pre-tDCS RT as an interacting (moderating) factor; similarly, the BK score was used for analyses of arm kinematics. Results showed that there were no impacts of the complexity of the task on RT. For both tasks, RT was significantly shorter following the application of anodal tDCS over SMA and M1, but this was moderated by pre-tDCS RT such that the participants with slower RT in pre-tDCS testing sessions benefited the most from stimulation (Figure 2). In SAS trials, neither task showed pre- and post-test RT effects of tDCS; however, SAS trials where a startle reflex was present (SCM+ trials) were initiated faster than the trials without a startle reflex (SCM–), but again, this was moderated by pre-tDCS RT such that the participants with slower RT in pre-tDCS testing sessions showed the largest SCM+/SCM– differences (Figure 3). Overall, kinematic variables for the elbow extension task were related to upper limb BK score, such that higher BK scores showed increased deficiencies in movement in terms of speed and displacement, but there were no substantial effects of anodal tDCS (either over SMA or M1) on any of the kinematic variables. On the other hand, the presentation of a SAS did lead to improvements in peak velocity, time-to-peak velocity, and time-to-peak displacement (Figures 4A,B, 5B). Overall, these results support the suggestion that response preparation is intact in individuals with PD, and thus indicate that anodal tDCS led to increased activation in structures associated with response initiation in both RT tasks; however, this effect was strongest in individuals that displayed slower RTs in the pretest. Finally, the improved response speed observed in SAS trials indicates that the increased activation provided by the startle reflex led to an overall decrease in bradykinetic symptoms.

### Impact of tDCS on RT and the StartReact Effect

The effect of anodal tDCS applied over the SMA or M1 on control trial RT was not the same for all individuals (Figure 2). In particular, it appears that anodal tDCS had little to no impact for those who had the shortest (fastest) RTs during pre-tDCS testing blocks, whereas anodal tDCS led to an increasingly greater RT benefit for those with longer (slower) pretest RTs. This effect led to all individuals exhibiting similar RTs following tDCS over M1 and SMA. This pattern of results suggests that both SMA and M1 stimulation improved RT for both tasks in the slower-reacting individuals.

The SMA is involved in many aspects of movement-related processing, including playing a particularly important role in the preparation and initiation of voluntary movements (15, 19, 50, 51). Previous work in healthy populations has demonstrated improvements in RT of upper-limb tasks, following the application of tDCS to the SMA, and these effects have been attributed to the upregulation of cortical structures involved in response preparation and initiation processes (21, 52). It is possible that, in the present study, premotor RT was improved in slower-reacting participants because SMA stimulation led to increased excitability of cortical areas associated with the preparation and/or initiation of each task. Furthermore, this speeding effect of tDCS on premotor RT appears to have been limited to those with the most pronounced bradykinesia, presumably underpinned by greater hypoactivation in the targeted structures. However, it should be noted that no direct measurement of changes in neural excitability was obtained, and thus, it can only be stated that the stimulation parameters led to the measured or observed behavioral changes. Further research would be needed to strengthen our understanding of the precise locus of neural changes induced by tDCS.

In addition to changes induced by tDCS applied over the SMA, results of the present study indicate that anodal tDCS can also produce significant changes in the level of activation in structures underlying response initiation processes when applied over M1. This result supports previous studies that have investigated the effects of tDCS applied over M1 for individuals with PD. For instance, in one study, it was reported that individuals with PD had faster RT after a single session of anodal tDCS (1 mA, 20 min) over M1 and that the motor improvements observed were likely due to increases in cortical excitability, compensating for the underactive pallido-thalamo-cortical drive that is a characteristic of PD (12). Moreover, another study showed that applying anodal tDCS (2 mA, 20 min) over the motor and premotor areas (which may have included stimulation of both M1 and SMA) over eight sessions led to improvements in bradykinesia, as assessed by sequential upper-limb movements (24).

The inclusion of SAS trials in the present study may provide some insight into the processes influenced by anodal tDCS that resulted in decreased RT in the slower participants with PD. When a SAS is presented in simple RT tasks, it can act as a synthetic trigger for a highly prepared response (42). One of the hypothesized mechanisms underlying this effect is that a startling stimulus directly increases activation in the structures responsible for voluntary response initiation *via* a subcortically mediated ascending pathway. In this way, the cortically stored response can be triggered without the usual cortical processing (37). RT facilitation following a SAS has also been observed in individuals with PD, suggesting that motor preparatory processes and pathways are not generally impacted (30, 32). For example, Fernandez-Del-Olmo et al. (32) investigated the effects of SAS by comparing a group of healthy individuals to those diagnosed with PD using a simple wrist flexion RT task and found that the StartReact effect for the upper limb movement was present in both groups. Additionally, Carlsen et al. (30) used a StartReact paradigm in individuals with PD and found that the startle-triggered RTs were not different whether the participants were “ON” or “OFF” anti-parkinsonian medication (30). Because the SAS only triggers responses that are sufficiently prepared (28), these results suggest that central processes involved in motor preparation are intact for individuals with moderate PD and that it is more likely that deficits in initiation processes are responsible for slowed RT in PD (30). In the present study, there was a large difference in pre-tDCS control trial RT between the fastest and slowest responders (~150 ms; see Figure 2). In contrast, there was very little difference in RT as a function of pre-tDCS RT on trials where a startle reflex was observed (SCM+; Figure 3). This result supports previous findings that preparatory activation was likely unimpaired in all individuals with PD and that decreased activation related to initiation processes likely underlies the RT deficits observed.

Interestingly, the RT results of the present study contrast with a previous report from our laboratory using a similar task, in which no RT differences were found in individuals with PD, following anodal tDCS applied over the SMA (25). One major difference between the experimental design of the previous study and the present experiment is that a StartReact testing paradigm was included within the present experimental protocol. The startle reflex arises from short latency activation of brainstem structures, such as the reticular formation (53), resulting in startle reflex-related activation conducted at various levels of the spinal cord *via* the reticulospinal tract. In addition, the startle reflex may cause ascending activation *via* projections from the pontine reticular formation to the thalamus. This increased activation of the thalamus may provide the required input to trigger the motor program of the prepared movement (37). Thus, in the present study, the addition of the SAS to the testing paradigm may have introduced increased activation to higher brain structures in SAS trials, leading to a greater and more sustained increase in activation in the brain areas associated with response initiation—even on non-SAS trials. This may have, in turn, led to a greater influence of tDCS on the same initiation-related structures, and, thus, the observed improvements in RT. However, this effect appeared to be most pronounced in participants with slower pre-test RTs. As such, heterogeneity in bradykinesia between participants my have masked any effect of tDCS on RT in previous studies. A direct comparison between experimental designs (anodal tDCS over SMA with SAS and without SAS) would be necessary to support this hypothesis.

### Kinematic Measures

For the elbow extension task, it was noted that individuals with higher BK scores executed the elbow extension more slowly, exhibiting lower peak velocity, longer time-to-peak velocity, and longer time-to-peak displacement than individuals with lower BK scores (Figures 4A,B, 5B). However, the application of tDCS did not lead to any significant pre-post differences in any of the kinematic measures in the elbow extension task, irrespective of the tDCS session. These results contrast those from a previous experiment using a similar task, in which improvements in movement time and time-to-peak displacement were observed following 10 min of anodal tDCS over SMA (25). One possible explanation for this difference could involve the limb used to perform each task. In the previous experiment (25), all the participants used their right limbs regardless of whether it was their most or least-affected side. In the present study, all the participants used their least-affected limbs to perform all the tasks (Table 1), which may have moderated the effects of tDCS on kinematic variables.

On the other hand, a significant effect of acoustic stimulus condition was seen on movement kinematics in that (overall) the movements were performed faster on startle trials. Specifically, peak velocity was higher, and time-to-peak velocity was shorter when the participants were startled (Figures 4A,B), and presenting a SAS also led to larger peak displacement (Figure 5A) and shorter time-to-peak displacement (Figure 5B). Previous studies involving SAS in healthy individuals have indicated that movement kinematics of responses triggered early in startle trials are largely unaffected, although some increases in initial force and peak displacement of the movement are sometimes observed (37). In the present experiment, the SAS promoted a faster movement execution, resulting in a decrease in movement time (time between the movement onset and final position) and time-to-peak displacement (time between the movement onset and peak displacement). These results mirror those reported previously in a study using a StartReact paradigm to investigate bradykinesia in individuals with PD (30). This previous study reported a decrease in bradykinetic symptoms in addition to shorter RT latencies in the SAS trials compared to control trials. Improved upper limb kinematics on SAS trials in individuals with PD has been previously explained by the SAS acting to normalize inappropriately small task-related EMG amplitude *via* increased activation from the startle reflex itself (30). Here, a similar result was seen, supporting the suggestion that centrally mediated EMG output magnitude deficits are the root cause of slowed movements in individuals with PD.

### Clinical Measures of Bradykinesia

In the present experiment, analysis of the BK score (upper limb function) showed no significant differences attributable to tDCS. This result indicates that, although tDCS did lead to RT improvements, it did not result in any specific clinical changes in bradykinesia as measured by the items selected from the UPDRS III. This lack of clinical improvement attributable to tDCS contrasts with previous work where tDCS was applied over M1 in an OFF-medication state, and improvements were seen in the total score of the UPDRS III (12). This result is also at odds with results showing a significant improvement in an upper-limb sequential movement test that was observed when tDCS was applied over the prefrontal cortex in the OFF-medication state (24). These differences between studies could be explained by the fact that the participants were tested during ON-medication states in the present study as compared to off-medication states in the aforementioned studies, and it is thus likely that the motor symptoms were already somewhat ameliorated by medication.

Although the UPDRS III is the gold standard to assess motor symptom severity in PD, it is possible that, because of the relatively small sample size in the present study and the variability in UPDRS III scores (see Table 1), the items selected to measure upper limb bradykinesia symptoms may not have been sensitive enough to detect small changes. Indeed, it has been previously argued that the UPDRS III has limited resolution for detecting small effects, and, thus, a large number of the participants are required to adequately power an experimental design investigating changes in the UPDRS III (54, 55). Furthermore, it has been suggested that the UPDRS as a whole is a relatively subjective assessment method, and research should strive to incorporate additional objective measures of bradykinesia when assessing overall motor dysfunctions associated with PD (55). Because anodal tDCS resulted in improvements to control trial RT in the particularly slow-reacting and slow-moving individuals, but these changes cannot be attributed to improved preparation, we argue that RT may constitute a more sensitive measure of bradykinesia related to response initiation.

### Limitations

The results from the present study should be considered in light of the following limitations. First, although sufficiently powered to detect significant differences in the dependent variables, the results of the study were collected from a small sample and without a control group. While a comparison between the control participants and the individuals with PD was not the primary goal of this research project, future work could aim to recruit age-matched controls to further assess the impact of anodal tDCS on motor areas. Second, a complete medication history was not recorded for individual participants as we were not comparing ON/OFF medication states. While testing ON-medication offers a more applied approach to understanding the benefits of anodal tDCS on individuals diagnosed with PD, we cannot exclude the possibility that some effects attributed to tDCS may be associated with medication. Third, in the present study, tDCS was applied for 10 min and removed prior to reassessing task performance, providing insight into the benefits of tDCS on subsequent movements; however, it is possible that concurrent online tDCS may have other effects. Future work could investigate the impact of concurrent online tDCS with task performance for further insight into using tDCS as a supporting therapeutic intervention. In addition, although there was a minimum of 48 h between any participant taking part in different tDCS sessions, we cannot fully exclude the possibility that there may have been some carry-over effect from the previous stimulation session, although this interval does fall within the suggested guidelines (41). Fourth, while we recognize that cognitive function can be impacted in some aggressive cases of PD, cognitive function was neither assessed prior to testing nor used as an exclusion criterion (e.g., *via* a Montreal Cognitive Assessment). The motor tasks used in the present experiment were both single actions (either finger flexion or elbow extension) and involved few instructions (a simple RT task); however, the results of the study should be considered with the caveat that not all the participants may have strictly adhered to instructions to react as quickly and accurately as possible. Furthermore, while the tasks used in the present experiment were both simple RT tasks, the performance of the tasks was always conducted in the same order (the button-press task followed by the elbow-extension task). It is possible that the results were impacted by this lack of randomization; however, since a small number of trials were performed by each participant, we do not expect that fatigue and/or practice had a large impact on the results. In addition, there were no kinematic measures available for the button-press task, which limited the available comparisons between the tasks. Future work could consider adding an accelerometer to the button press or to the hand to gain further insight into differences in kinematic measures between the two simple RT tasks. Lastly, while all the participants recruited for the present study were right-handed, many participants were tested with their left (non-dominant) limbs as they reported their right limbs as the most affected. The least-affected limb was assessed in order to limit tremor interference with the EMG signal; however, we cannot exclude the possibility that limb dominance may have impacted the results.

## Conclusion

The present study used two upper limb tasks to examine premotor RT, kinematic variables, and measures of bradykinesia in individuals with PD before and after anodal tDCS of the SMA and M1. Results showed that anodal tDCS applied over the SMA and M1 for 10 min improved premotor RT, but only in the participants with slower responses (RT) in pre-tDCS testing. Therefore, it is likely that the individuals with PD who may have greater impairment of motor initiation processes would see increased benefits from anodal tDCS applied over the SMA or M1. When a SAS replaced the typical go-stimulus, premotor RT and response kinematics were also improved for both groups, supporting previous reports that motor preparation processes are relatively intact in individuals with PD. As such, the RT improvements observed following anodal tDCS and a SAS can most likely be attributed to increased activation associated with response initiation processes.

## Data Availability Statement

The raw data supporting the conclusions of this article will be made available by the authors, without undue reservation.

## Ethics Statement

The studies involving human participants were reviewed and approved by the University of Ottawa Health Sciences and Science Research Ethics Board. The patients/participants provided their written informed consent to participate in this study.

## Author Contributions

AK, JN, and AC conceived and designed the study. CS, AK, and JL acquired, marked, and collated the data. CS and AC analyzed and interpreted the data and drafted the manuscript. All the authors contributed to the critical revision and approval of the final version of the manuscript.

## Funding

This work was supported by the Natural Sciences and Engineering Research Council of Canada [Grant No. 2017-04717] and the Ontario Ministry of Research, Innovation and Science [Grant No. ER14-10-133]. The funding sources were not involved in the design or execution of the research, analysis, interpretation of the results, or writing of the manuscript.

## Conflict of Interest

The authors declare that the research was conducted in the absence of any commercial or financial relationships that could be construed as a potential conflict of interest.

## Publisher's Note

All claims expressed in this article are solely those of the authors and do not necessarily represent those of their affiliated organizations, or those of the publisher, the editors and the reviewers. Any product that may be evaluated in this article, or claim that may be made by its manufacturer, is not guaranteed or endorsed by the publisher.
